# A thermosensitive gel matrix for bioreactor-assisted in-cell NMR of nucleic acids and proteins

**DOI:** 10.1007/s10858-023-00422-7

**Published:** 2023-09-09

**Authors:** Matej Dzurov, Šárka Pospíšilová, Michaela Krafčíková, Lukáš Trantírek, Lucy Vojtová, Jan Ryneš

**Affiliations:** 1 CEITEC – Central European Institute of Technology, Brno University of Technology, Purkyňova 656/123, Brno, 612 00 Czech Republic; 2 CEITEC – Central European Institute of Technology, Masaryk University, Kamenice 753/5, Brno, 625 00 Czech Republic; 3 National Centre for Biomolecular Research, Masaryk University, Kamenice 753/5, Brno, 625 00 Czech Republic; 4 Institute of Biophysics, Czech Academy of Sciences, Královopolská 135, Brno, 612 65 Czech Republic; 5 Bijvoet Centre for Biomolecular Research, Utrecht University, Padualaan 12, Utrecht, 3584 CH The Netherlands

**Keywords:** PLA-PEG-PLA, Thermosensitive hydrogel, Cell immobilization, Bioreactor, In-cell NMR

## Abstract

Introducing the flow through the bioreactor has revolutionized in-cell NMR spectroscopy by prolonging the measurement time available to acquire spectral information about biomacromolecules in metabolically active cells. Bioreactor technology relies on immobilizer matrices, which secure cells in the active volume of the NMR coil and enable uniform perfusion of the growth medium, supplying fresh nutrients to the cells while removing toxic byproducts of their metabolism. The main drawbacks of commonly used matrices include the inability to recover intact cells post-measurement for additional analyses and/or requirements for specific operating temperatures. Here, we report on the development and characterization of a set of thermosensitive and nontoxic triblock copolymers based on poly(D,L-lactide)-*b*-poly(ethylene glycol)-*b*-poly(D,L-lactide) (PLA-PEG-PLA). Here, we show for the first time that these copolymers are suitable as immobilizer matrices for the acquisition of in-cell NMR spectra of nucleic acids and proteins over a commonly used sample temperature range of 15–40 °C and, importantly, allow recovery of cells after completion of in-cell NMR spectra acquisition. We compared the performances of currently used matrices in terms of cell viability (dye exclusion assays), cellular metabolism (1D ^31^P NMR), and quality of in-cell NMR spectra of two model biomacromolecules (hybrid double-stranded/i-motif DNA and ubiquitin). Our results demonstrate the suitability and advantages of PLA-PEG-PLA copolymers for application in bioreactor-assisted in-cell NMR.

**Supplementary Information:**

The online version contains supplementary material available at 10.1007/s10858-023-00422-7.

## Introduction

In-cell NMR spectroscopy delivers unique information about the structure and interactions of biomolecules in their native environment inside living cells and has been recently thoroughly reviewed in (Theillet 2022; Luchinat and Banci 2022). The original in-cell NMR approach (Serber et al. 2001; Inomata et al. 2009) requires concentrating a large number of cells in a small volume of NMR coil, which results in nutrient depletion from the surrounding media. Cellular anaerobic metabolism then leads to the evolution of byproducts and acidification of the sample, which eventually results in higher cell mortality. Therefore, the in-cell NMR measurements using this setup have been time-limited to minimize the effects of the changing environment within the sample.

A bioreactor is a specialized flow-through NMR cuvette that allows a continuous supply of fresh medium to the cells within in-cell NMR samples. Currently, bioreactors are commonly used to avoid problems accompanied by compromised metabolic activity and cell mortality during prolonged in-cell NMR measurements (Gmati et al. 2005; Sharaf et al. 2010; Kubo et al. 2013; Inomata et al. 2017; Breindel et al. 2018, 2020; Carvalho et al. 2019; Cerofolini et al. 2019). The bioreactor can extend the in-cell NMR acquisition window up to 72 h (Cerofolini et al. 2019). In a bioreactor setup, the cells are immobilized in a permeable gel matrix to prevent washout of cells away from the active volume of the NMR coil by the media flow. Various gel matrices, including alginate, Mebiol® gel, and low-melting agarose, i.e., polymers with chemically or temperature-regulated sol-gel transitions, have been used to immobilize cells in bioreactor systems (Kubo et al. 2013; Luchinat et al. 2020; Breindel et al. 2020).

Alginate is an example of a polymer with ion-induced gelation. In the presence of divalent cations (such as Ca^2+^), linear chains of alginate form crosslinks, and alginate forms a gel. The Ca^2+^-crosslinked alginate matrix is biocompatible and nontoxic, and it has been used for decades to immobilize cells in 2D and 3D cultures for various purposes (Andersen et al. 2015). There are two main advantages of using alginate as an immobilization matrix in bioreactor-assisted in-cell NMR. First, alginate-entrapped cells can be released following in-cell NMR spectra acquisition by chelation of Ca^2+^ ions with trisodium citrate without compromising cell viability (Smidsrød and Skjåk-Braek 1990; Lee et al. 1991). Second, alginate can expand as the cells grow (Breindel et al. 2020). Its use might therefore be advantageous in cases when cells grow and divide during the in-cell NMR experiment, such as in the case of bacterial cells. However, using alginate to prepare in-cell NMR samples with a uniform density of alginate-entrapped cells is challenging, as the production of alginate beads of uniform size often requires special instrumentation (Sharaf et al. 2010; Inomata et al. 2017; Breindel et al. 2020). Additionally, culture media often contain sodium and phosphate ions that may destabilize Ca^2+^-crosslinked alginate gel, adversely affecting its mechanical strength (Smidsrød and Skjåk-Braek 1990).

Unlike alginate, preparing of NMR samples in polymers with temperature-induced gelation does not require special instrumentation. Low melting agarose is currently the most widely used carrier of cells for bioreactor experiments (Breindel et al. 2018; Carvalho et al. 2019; Luchinat et al. 2020; Barbieri and Luchinat 2021). Agarose is biocompatible and forms a gel resistant to media commonly used for cell cultivation (Renn 1984; Fernández-Cossío et al. 2007). Hydroxyethyl modification of agarose decreases the gelling point to 8–17 °C (SeaPrep® Agarose, Lonza Rockland, Inc.). The resulting gel is then stable at 37 °C, which allows the preparation of the NMR sample at temperatures that are not harmful to the cells. However, a melting point of 50 °C and above does not allow the recovery of intact/viable cells from the gel. Thus, the immobilized cells from in-cell NMR measurements cannot be used for subsequent analyses and viability assays.

Nevertheless, cell recovery can be achieved with gels with reverse temperature dependence, where the gel is a liquid at low temperatures and gels when heated. The only commercially available gel with these properties that has been successfully employed in a bioreactor-assisted NMR measurement is Mebiol® gel (Cosmo Bio Co., Ltd.), a copolymer of poly(N-isopropyl acrylamide) and poly(ethylene glycol) with a temperature-dependent reversible transition, being liquid at 4 °C and solid at 37 °C. The gel is biocompatible and nontoxic and has been used for the growth and differentiation of stem cells in 2D and 3D cultures (Lin et al. 2017; Rodrigues et al. 2017). However, some applications of in-cell NMR, such as acquiring ^1^ H spectra of nucleic acid or using cell lines other than human cells (e.g., insect cells), require measurement at a temperature of approximately 20 °C. Mebiol® gel is in a liquid state at this temperature, which does not allow cell immobilization.

In the last few decades, thermosensitive copolymers consisting of poly(lactic*-co-*glycolic acid)*-b-*poly(ethylene glycol)*-b-*poly(lactic*-co-*glycolic acid) (PLGA-PEG-PLGA), commercially known as ReGel® and OncoGel®, have been used in the localized and controlled drug delivery of various therapeutic molecules due to their tunable biodegradability and gelation at physiological temperatures (Elstad and Fowers 2009; Feng et al. 2014; Wang et al. 2017; Perinelli et al. 2019; Lysáková et al. 2022). In an aqueous environment, amphiphilic molecules of PLGA-PEG-PLGA self-assemble into flower-like micelles, which undergo reversible intermicellar bridging induced by an increase in temperature. This leads to physical gelation and the formation of a permeable gel environment with high water content (Gong et al. 2013). In PLGA copolymers, the rate of degradation is a consequence of the D,L-lactide (LA)/glycolide (GA) molar content in the copolymer structure, where a 50:50 molar ratio has the fastest degradation rate. In addition, the degradation itself is accompanied by the release of lactic and glycolic acid molecules, leading to a decrease in pH (Makadia and Siegel 2011; Oborná et al. 2016).

Here, we report the development and characterization of novel thermosensitive tri-block PLA-PEG-PLA copolymers consisting of only poly(D,L-lactide) (PLA) and poly(ethylene glycol) (PEG) that behave as a liquid at low temperatures and form a hydrogel upon heating. Without the presence of glycolide (GA) in the copolymer, the hydrolytic degradation behavior and decrease in pH are considerably lower without otherwise interfering with the properties of the hydrogel. The sol-gel transition of these copolymers is dependent on the chemical structure, ratios of hydrophobic poly(D,L-lactide) to hydrophilic poly(ethylene glycol) subunits, and concentration of the solution (Hoare and Kohane 2008). In this research, we thoroughly compared the performances of commercially available immobilizer matrices, SeaPrep® agarose and Mebiol® gel, with two types of PLA-PEG-PLA copolymers synthesized at our facilities. We show that these copolymers are nontoxic to cells and have properties compatible with a bioreactor setup. In addition, we demonstrated the ability to tune the copolymer composition concerning monomer feed ratios, in turn achieving desirable thermoresponsive characteristics, enabling the acquisition of in-cell NMR spectra at 20 and 37 °C and release of immobilized cells following in-cell NMR measurements.

## Materials and methods

### Synthesis and purification of LEL triblock copolymers

The synthesis of the tri-block copolymers denoted LEL-20 and LEL-37 was carried out via conventional ring-opening polymerization (ROP) of 0.102 mol D, L-lactide (Polysciences Europe GmbH, Germany) with 0.0043 mol poly(ethylene glycol) (PEG, *M*_*n*_ = 1000 g·mol^− 1^ and 1500 g·mol^− 1^, Merck KGaA, Germany) in the presence of 0.001 mol stannous octoate (Merck KGaA, Germany) as a catalyst. The reaction proceeded on the Schlenk line without a solvent, i.e., in the melt, under an inert nitrogen atmosphere according to the modified procedure described by Michlovská et al. 2010. Subsequently, the copolymer melt was purified from unreacted monomers and catalyst. The single purification step required the dissolution of the copolymer in water and subsequent heating of the solution to 80 °C, which caused the copolymer to precipitate from the solution, leaving unreacted monomers and the catalyst in the solution. The residual water with impurities was discarded, and the purification procedure was repeated 3 times in total. The final purified product was freeze-dried (Martin Christ EPSILON 2-10D, Germany) until constant weight.

### Gel permeation chromatography (GPC)

The number-average molecular weight (*M*_*n*_), mass-average molecular weight (*M*_*w*_), and polydispersity index (PDI = *M*_*w*_*/M*_*n*_) of the copolymers were measured using the gel permeation chromatography (GPC) method on a Waters Alliance e2695 GPC system (Waters Corporation, Milford, MA, USA) equipped with an Agilent PLgel 5 μm MIXED-D column at 65 °C and a refractive index detector at 40 °C, with dimethylformamide (DMF, 99.8%, Biosolve BV, The Netherlands) with 10 mM LiCl (≥ 99%, Sigma‒Aldrich, The Netherlands) as the eluent at a flow rate of 1 ml/min against linear poly(ethylene glycol) standards. The data were evaluated using Empower 3 Software 2010. Approximately 3 mg of copolymers were dissolved in 1 ml of DMF + 10 mM LiCl eluent, filtered prior to measurement, and analyzed.

### Preparation of LEL tri-block copolymer solutions

The copolymers were weighed into vials and dispersed in Dulbecco’s modified Eagle medium (DMEM, Sigma‒Aldrich, USA) at the desired concentration to buffer the acidic reaction and ensure cellular survival in a hydrogel matrix. The dissolution of copolymers was carried out on a magnetic stirrer at 300–500 rpm for 3 days at a decreased temperature in the range of 4 to 12 °C until a completely homogeneous solution was obtained. For in-cell NMR experiments, LEL-20 and LEL-37 concentrations of 25 and 15 w/w %, respectively, were used.

### Test tube inversion method (TTIM) and pH measurement

The sol-gel phase transitions of tri-block copolymer solutions were investigated visually using the test tube inversion method on the thermal block (DITABIS AG, Germany). The range for the experiments was set from 10 to 40 °C with a 2 °C temperature increment and an equilibration time of 5 min. The sol-gel transition was evaluated based on the criterion of flow. When the solution exhibited flowing characteristics, it was determined to be in the solution state. When the solution stayed in the bottom of the test tube after inversion, it was concluded that the solution was in a gel state. The pH value was obtained by reading the laboratory pH meter (Verkon, Czech Republic) after 1 min of equilibration.

### Rheological analysis

Rheological properties of copolymer dispersions were determined using a controlled stress rheometer (Discovery HR-2, TA Instruments, USA) equipped with steel plate-plate geometry (40 mm) with a solvent trap and a Peltier plate temperature system. The temperature sweep measurements were carried out within the linear viscoelastic region of the material with a geometry gap set to 200 μm applying a constant 1 Hz frequency (6.28 rad·s^− 1^), oscillation strain of 1%, temperature ramp of 1 °C per minute and a temperature range from 10 to 40 °C.

### DNA oligonucleotide preparation

The DNA oligonucleotides 5’-FAM-labeled and unlabeled 5’-GCT TCT AGT CAA TCC CCC CTC CCC CCT TCC CCC CTC CCC CC-3’ and unlabeled 5’-TTG ACT AGA AGC-3’ were purchased from Generi Biotech (Czech Republic). The nonlabelled oligonucleotides were first dissolved in H_2_O and subjected to standard n-butanol precipitation to remove contaminants from the solid-state synthesis as described by Viskova et al. 2019. Briefly, 30 ml of n-butanol (≥ 99%, Sigma‒Aldrich, USA) was added to ~ 1 ml of an aqueous solution of the DNA oligonucleotide. The resulting mixture was then vigorously shaken for 10 minutes and centrifuged at 30 000×g at 4°C for 1 hour. After centrifugation, the supernatant was removed, and upon drying, the pellet was redissolved in 1 ml of H_2_O. The DNA concentration was determined spectrophotometrically, and the strands were mixed at a ratio of 1:1.1 and annealed by incubation at 95°C for 5 minutes, followed by gradual cooling to room temperature. To form the 5’-FAM-labeled double-stranded DNA, the reverse strand was mixed with the FAM-labeled forward strand in the same ratio as mentioned above. The samples were then annealed for 10 min at 37 °C.

### Cell culture

HeLa cells (Sigma‒Aldrich, USA) and HEK293T cells (ATCC, USA) were cultured in Dulbecco’s modified Eagle’s medium (DMEM) (Sigma‒Aldrich, USA) supplemented with 10% heat-inactivated fetal bovine serum (FBS) (HyClone, GE Life Sciences, USA) and penicillin‒streptomycin solution (P/S) (100 units penicillin and 0.10 mg streptomycin/ml) (Sigma‒Aldrich, USA) under a 5% CO_2_ atmosphere at 37 °C. At 80–90% confluency, the cells were passaged by washing with Dulbecco’s phosphate-buffered saline (DPBS) (Sigma‒Aldrich, USA) and by harvesting with 0.05% trypsin and 0.02% EDTA (Sigma‒Aldrich, USA) in 1 × DPBS.

### Sample preparation for DNA in-cell NMR spectra acquisition

A total of 8 × 10^7^ pelleted HeLa cells were resuspended in 2 ml of electroporation buffer (140 mM sodium phosphate, 5 mM KCl, 10 mM MgCl_2_, pH = 7.0) containing 400 µM DNA and 10 µM FAM-labeled DNA. The cell suspension was divided into five 4-mm electroporation cuvettes (Cell Projects, UK). After a 5-minute incubation on ice, electroporation was conducted with a BTX-ECM 830 system (Harvard Apparatus, USA) using two square-wave pulses (100 µs/1000 V; 30 ms/350 V) separated by a 5-second interval. After electroporation, the cells were incubated for 2 min at room temperature. The suspension from electroporation cuvettes was transferred to a centrifugation tube containing 10 ml of DMEM. The cells were pelleted by centrifugation at 200×g for 5 min and washed with 10 ml of DMEM. An aliquot of cells (~ 1 × 10^5^) was used for flow cytometry analysis to determine cell survival and transfection efficiency. For ^1^ H NMR DNA spectra acquisition, the cell pellet was mixed either with 2 w/w % SeaPrep® agarose in DMEM at 37 °C or with 25 w/w % LEL-20 in DMEM at 4 °C, both in a v:v ratio of 1:1 to obtain a final volume of 500 µl.

### Sample preparation for protein in-cell NMR spectra acquisition

HEK293T cells were transfected with plasmid pHL-sec, bearing the coding sequence for human ubiquitin (Tanaka et al. 2019), using poly(ethylenimine) (PEI) as described in Banci et al. 2013. Briefly, 25 µg of plasmid DNA was mixed with 50 µg of PEI in 5 ml of ^15^ N-labeled BIOEXPRESS-6000 media (Cambridge Isotope Laboratories, UK). The culture medium in a subconfluent 75 cm^2^ culture flask was replaced with the transfection mixture, followed by the addition of 15 ml BIOEXPRESS-6000 medium with 2% FBS. The cells were incubated for 48 h without a medium change to express the protein. For the acquisition of ^15^ N-labeled protein spectra, the cells were harvested with 0.05% trypsin and 0.02% EDTA, washed with DMEM, and centrifuged for 5 min at 200×g. To obtain a total volume of 500 µl, the cell pellet was mixed with 15 w/w % LEL-37 in DMEM or 10 w/w % Mebiol® gel in DMEM at a v:v ratio of 1:2 at 4 °C.

### Sample preparation for intracellular ATP monitoring

For monitoring of cell metabolic activity via ATP level determination from ^31^P NMR spectra, HeLa cells were harvested with 0.05% trypsin and 0.02% EDTA (Merck KGaA, Germany), washed with DMEM, and centrifuged for 5 min at 200×g. To obtain a total volume of 500 µl, the cell pellet was mixed with 4 w/w % SeaPrep® agarose (Lonza, Switzerland) in DMEM in a v:v ratio of 3:1 at 37 °C, 10 w/w % Mebiol® gel (Cosmo Bio, USA) in DMEM 1:1 at 4 °C, 15 w/w % LEL-37 in DMEM 2:1 at 4 °C and 25 w/w % LEL-20 DMEM 2:1 at 4 °C.

### Bioreactor setup

A PEEK capillary with a 0.75 mm inner diameter was filled with the mixture of polymer with cells. To solidify, the samples in SeaPrep® agarose were incubated on ice for 10 min, the samples in Mebiol® gel and LEL-37 at 37 °C for 20 min, and LEL-20 at room temperature for 20 min. A thread of the gel was pushed with a syringe from the capillary into a 5 mm screw-cap NMR tube filled with bioreactor media: DMEM without NaHCO_3_ (Sigma‒Aldrich, USA), 10% D_2_O (Eurisotop, France), 70 mM HEPES (Sigma‒Aldrich, USA), ZellShield (Minerva Biolabs, Germany). The NMR cuvette with the sample was connected to a tubing system ensuring media flow. The flow of medium was driven by HPLC pump (ECP2010, ECOM, Czech Republic) from a reservoir incubated in a water bath at 37 °C or at room temperature, through vacuum degassing system (DG 4014, ECOM, Czech Republic), to the bottom of the NMR cuvette via a glass capillary connected to the inlet tubing. The fresh medium was flowing up through the sample, thereby displacing the nutrient-depleted medium, which was drained via the outlet tubing connected to an orifice in the cuvette lid. The flow rate was set to 50 µl/min.

### Release of cells from thermosensitive matrices

Gel threads with cells were transferred into a Falcon tube, containing 25 ml of DPBS at 4 °C. To dissolve the matrix, the tube was incubated for 10 min on a rotator at 4 °C. Subsequently, the suspension was filtered through 70 μm cell strainer (Corning, USA) to remove undissolved pieces of gel.

### 1D ^1^ H and ^31^P NMR experiments

1D ^1^ H NMR spectra for copolymer characterization and 1D ^31^P NMR spectra for ATP level measurement were recorded on a Bruker Avance NEO 500 MHz NMR (Bruker Corporation, Billerica, MA, USA) equipped with a 5 mm nitrogen-cooled dual (BB-^1^ H) cryoprobe (Prodigy). For the copolymer (LEL-20 and LEL-37) measurement, approximately 10 mg of sample was weighed on analytical balances and consecutively dissolved in 500 µl of deuterated chloroform (CDCl_3_). The sample was transferred into a 5 mm NMR cuvette and measured by a 1D ^1^ H zg pulse sequence with 128 scans at 20 °C. The number-average molecular weight was established from the ratio of PEG signal intensity (-CH_2_-O-CH_2_-) from δ = 3.34–3.84 ppm to the PLA (-CH-) signal from δ = 5.29–5.05 ppm. For ATP level measurements, 500 µl of a sample (see the NMR sample preparation) was transferred into a 5 mm NMR cuvette and measured by a 1D ^31^P zgdc pulse sequence with 4 × 512 scans at 20 °C (LEL-20 and SeaPrep® agarose) and 37 °C (LEL-37 and Mebiol® gel) with a bioreactor system. The obtained spectra were processed with the QSIN (sine squared) function with the SSB parameter set to 2.2. The NMR spectra were processed, integrated, and analyzed using MNova v14.2.1 software (Mestrelab Research, Spain).

### In-cell 1D ^1^ H and 2D ^1^ H – ^15^ N NMR experiments

In-cell 1D ^1^ H NMR spectra for DNA and 2D ^1^ H – ^15^ N NMR spectra for protein investigation were recorded on a Bruker Avance NEO 950 MHz NMR (Bruker Corporation, Billerica, MA, USA) equipped with a 5 mm triple-resonance (^1^ H/^19^F-^13^ C-^15^ N) inverse cryoprobe with cooled ^1^ H and ^13^ C preamplifiers and a bioreactor system. A 1D ^1^ H JR-echo (1–1 echo) pulse sequence with zero excitation set to the resonance of water and the excitation maximum set to 13 ppm was used for the in-cell DNA measurement. Spectra were measured at 20 °C (LEL-20 and SeaPrep® agarose) with 5 × 1024 scans. Spectra were processed by the exponential apodization function with the line-broadening parameter (LB) set to 30 and baseline corrected. A 2D ^1^ H – ^15^ N SOFAST-HMQC pulse sequence was used for the in-cell protein measurement. Spectra were recorded at 37 °C (LEL-37 and Mebiol® gel) with five independent measurements consisting of 100 scans and 128 increments. The obtained spectra were processed by an exponential apodization function with the line-broadening parameter (LB) set to 10. The NMR spectra were processed and analyzed using Bruker Topspin 4.0 (Bruker Corporation, Billerica, MA, USA) and MNova v14.2.1 software (Mestrelab Research, Spain).

### Cell viability analysis using trypan blue staining

HeLa cells were embedded in the tested polymers and incubated in 1 ml of the bioreactor media (DMEM without NaHCO_3_ + 10% D_2_O + 70 mM HEPES + ZellShield) at room temperature. At the specified time points, aliquots of the samples were homogenized in 100 µl of DPBS, and 20 µl of Trypan blue (Sigma‒Aldrich, USA) was added. After 2 min of incubation, 20 µl of the suspension was transferred to a microscope slide, and pictures of three random areas of each sample were taken. From each image, an area containing at least 100 cells was selected, where blue (dead) and white (living) cells were counted. The percent viability was calculated as the number of living cells/total number of cells×100, and the results from the three areas were averaged.

### Flow cytometry analysis

Approximately 1 × 10^5^ of HeLa or HEK293T cells were resuspended in 200 µl of DPBS, and propidium iodide (Sigma‒Aldrich, USA) was added to a final concentration of 2.5 µg/µl. Samples were analyzed using a BD FACS Verse Cell Analyzer (BD Biosciences, USA). For identification of propidium iodide-positive cells, the excitation wavelength was set to 488 nm, and emission at 700 ± 54 nm was detected. To determine the DNA transfection efficiency, the FAM label was detected as emission at 572 ± 32 nm upon excitation at 488 nm. A total of 1 × 10^4^ cells were analyzed in each experiment. The results were processed with BD FACSsuite software (BD Biosciences, USA).

The membrane integrity of HeLa cells immobilized in thermosensitive copolymer gels was further tested under bioreactor conditions equipped with constant media flow. Threads of the cells embedded in Mebiol® gel, LEL-37, and LEL-20 were placed into an NMR bioreactor and incubated at 37 °C (Mebiol® gel, LEL-37) or 20 °C (LEL-20) at a media flow of 50 µl/min. As a control representing conditions without a polymer matrix, cells were incubated on dishes with nonadhesive surfaces in excess of the bioreactor media (instead of continuous flow) without a CO_2_-enriched atmosphere. After 4 h of incubation, the cells were isolated from the gel matrices and stained with propidium iodide (PI) to determine their membrane integrity using fluorescent flow cytometry as described previously.

### Confocal microscopy

After NMR measurement, HeLa cells were released from the immobilization matrices and washed with cold DPBS. Approximately 5 × 10^5^ cells were transferred to a 35 mm glass-bottom iBidi dish (Ibidi GmbH, Germany) precoated with 0.2% gelatin (Sigma‒Aldrich, USA) supplemented with 2 ml of DMEM containing 25 ng/ml SiR-DNA far-red dye (Tebubio, France) to visualize the cell nuclei. All microscopy images were obtained using a Zeiss Axio Observer. Z1 with an LSM 800 confocal unit, CO_2_ incubation chamber, and Plan-Neofluar 10×/0.30 AIR objective. Images were taken every 10 min in eight different positions during a 16-hour period in transmission mode with 640 nm excitation and emission detection at 650–750 nm. Confocal images were processed with ZEN Blue software (Zeiss, Germany).

## Results

### Synthesis and material properties of LEL copolymers

**Fig. 1:**
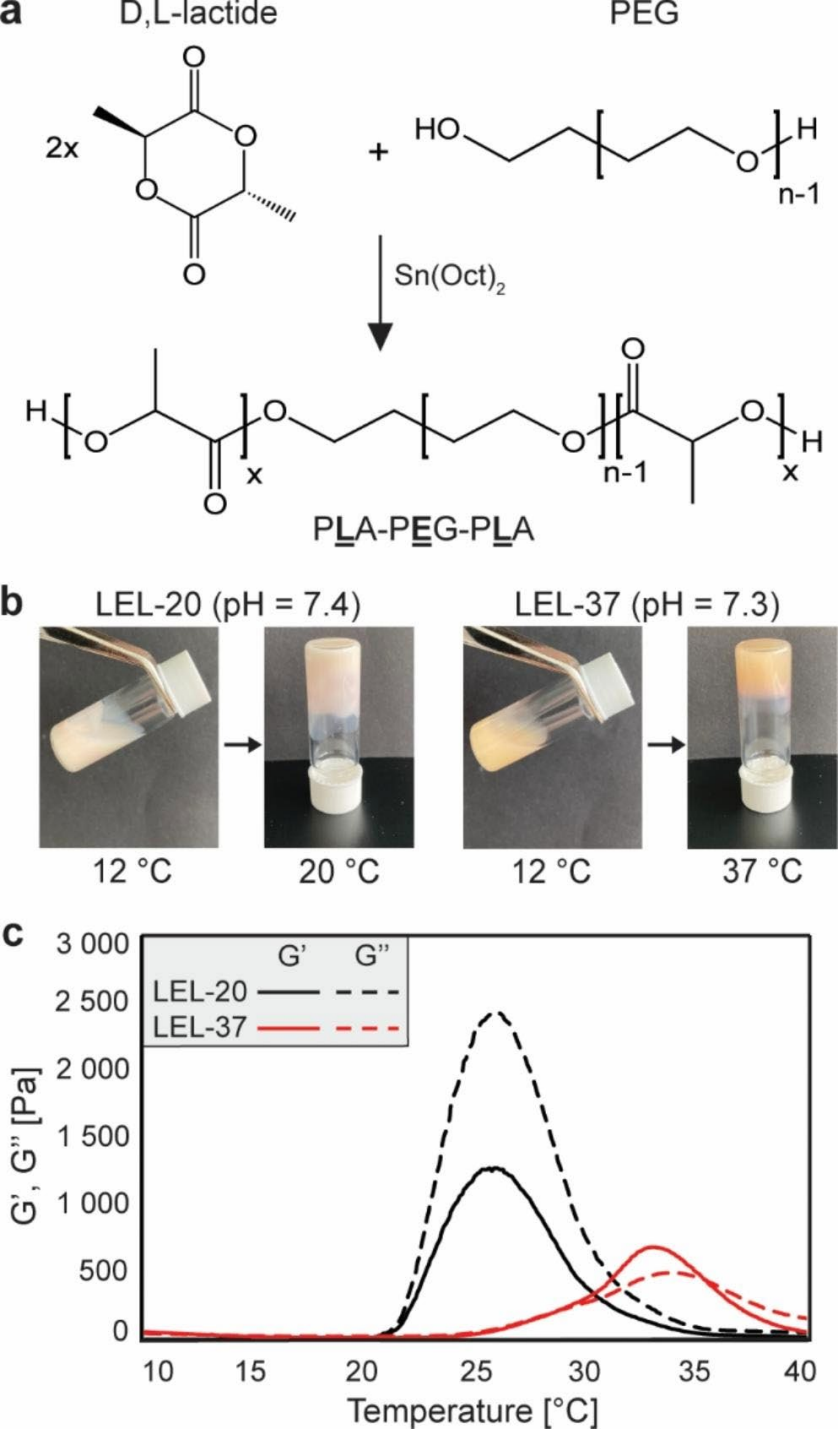
(**a**) Schematic representation of LEL copolymer synthesis. (**b**) pH values and test tube inversion of 25 w/w % LEL-20 and 15 w/w % LEL-37 copolymer solutions in DMEM with increasing temperature showing the occurrence of gelation at the desired temperature. (**c**) Temperature ramp with storage modulus – G’ and loss modulus – G’’ of 25 w/w % LEL-20 and 15 w/w % LEL-37 copolymer solutions in DMEM showing differences in material performance with different copolymer compositions

The synthesis of the tri-block copolymer termed LEL (from P**L**A-P**E**G-P**L**A) was carried out via ring-opening polymerization of D, L-lactide with poly(ethylene glycol) in the presence of stannous octoate as a catalyst (Fig. 1a). Two types of PLA-PEG-PLA copolymers that differ in the molecular weight of the PEG subunit were synthesized: LEL-20 containing 1000 g/mol PEG, intended for NMR measurements at 20 °C, and LEL-37, which contains 1500 g/mol PEG, intended for experiments at 37 °C (Table 1). GPC and ^1^ H NMR spectroscopy were used to characterize the average molecular weights (*M*_*n*_, *M*_*w*_) and PDI of the prepared copolymers (Table 1, Supplementary Fig. S1, S2, and Supplementary Table S1). The deviation of experimentally obtained molecular weights compared to theoretical calculations is a synthetic consequence of the 90% PLA monomer conversion and 100% PEG monomer conversion after 3 h of reaction time, as previously reported (Du et al. 1995). The low polydispersity indicates a uniform distribution of copolymer chains, which is necessary for the reproducibility of the material performance. GPC chromatograms and NMR evaluation are shown in the supplementary information (S1, T1, S2).

**Table 1:** Theoretical and experimental characterization of used LEL copolymers (PLA – block molecular weight, PLA/PEG – hydrophobic-to-hydrophilic block ratio, M_n_ – number-average molecular weight, M_w_ – weight-average molecular weight, PDI – polydispersity index)

		Theoretical			1D ^1^ H NMR		GPC		
Copolymer	PEG [g·mol^− 1^]	PLA [g·mol^− 1^]	*M*_*n*_ [g·mol^− 1^]	PLA/PEG	*M*_*n*_ [g·mol^− 1^]	PLA/PEG	*M*_*n*_ [g·mol^− 1^]	*M*_*w*_ [g·mol^− 1^]	PDI *M*_*n*_*/M*_*w*_
**LEL-20**	1000	3450	4450	3.45	4035	3.04	3931	4609	1.17
**LEL-37**	1500	3450	4950	2.30	4577	2.05	4444	5105	1.15

A tube inversion test confirmed the formation of a hydrogel network of 25 w/w % LEL-20 at 20°C and 15 w/w % LEL-37 at 37°C, and pH measurements showed that copolymer solutions were indeed in the desired physiological pH range (Fig. 1b). Further rheological analysis of 25 w/w % LEL-20 solution in DMEM confirmed the temperature-sensitive behavior with an increase in the elastic component - storage modulus (G’), starting at 18 °C and reaching an absolute maximum at 26 °C with G’ = 1200 Pa (Fig. 1c). The values of the viscous component – the loss modulus (G’’), were twice as high (G’’ = 2400 Pa) in comparison to the storage modulus – indicating possible phase separation. However, this phase separation phenomenon was not observed at temperatures below 30 °C, as seen with the inversion method (Fig. 1b). LEL-37 at a concentration of 15 w/w % exhibited slightly higher rheological moduli and appeared more viscous at low temperatures than LEL-20. LEL-37 showed gelation at 29 °C, reaching a maximum storage modulus of 680 Pa at 33 °C. Above 35 °C, phase separation occurs, and some of the water in the network is excluded from the hydrogel. However, again, the phase separation behavior of LEL-37 was not observed with the test tube inversion until temperatures reached above 40 °C. Differences in rheological data and the test tube inversion method are a consequence of the larger volume of copolymer solution used for test tube inversion (1 ml) compared to rheological measurements (0.25 ml) and the differences in temperature ramps of these methods (rheology – 1 °C per minute, TTIM – 2 °C step followed by 5 min equilibration).

### Impact of cell immobilization in LEL on viability and metabolic activity

**Fig. 2:**
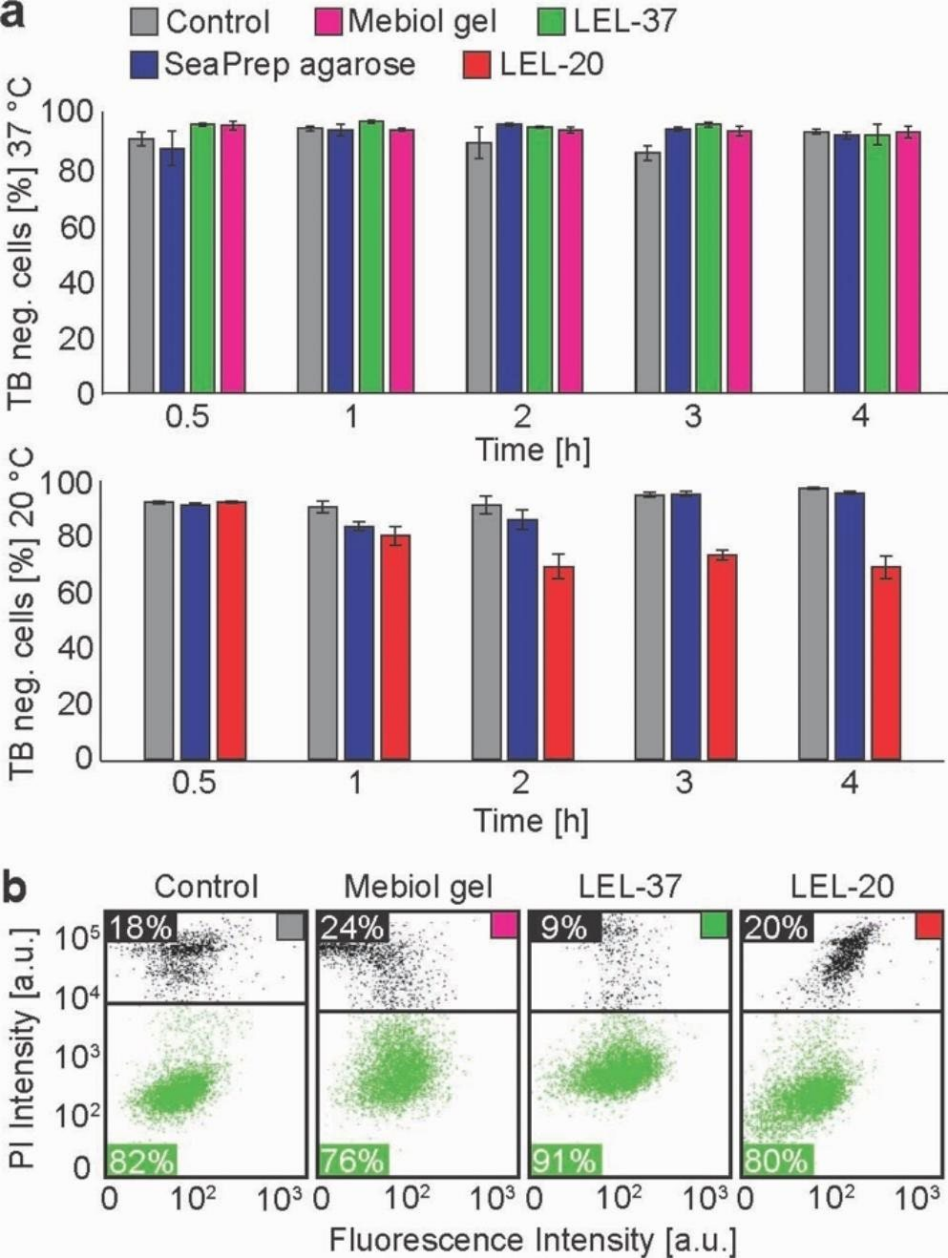
(**a**) Trypan blue (TB) staining viability assays at 37 °C and 20 °C of control HeLa cells (gray) and HeLa cells immobilized in SeaPrep® agarose (blue), copolymer LEL-20 (red), LEL-37 (green), and Mebiol® gel (magenta). (**b**) Flow cytometry live/dead (green/black) viability assay based on propidium iodide staining of HeLa cells released from the immobilization matrix after 4 h in bioreactor

To assess the performance of LEL as an immobilizing matrix for in-cell NMR applications, we compared the impact of LEL and other established matrices (Mebiol® gel and low-melting SeaPrep® agarose) on HeLa cell viability, determined via dye exclusion assays. To simulate the conditions in an NMR tube during in-cell spectra acquisition, samples were incubated in 1.5 ml Eppendorf tubes containing noncirculating bioreactor media. Since it is not possible to isolate the cells from SeaPrep® agarose for flow cytometry analysis, trypan blue (TB) staining (reporting on cell membrane integrity) and manual counting under the light microscope have been employed to allow comparison of the matrices. Following 4 h of incubation at 37 °C, the number of cells with compromised membrane integrity in pellet, SeaPrep® agarose, Mebiol® gel, and LEL-37 was comparable, with differences of less than 10% between individual samples (Fig. 2a). At 20 °C, Mebiol® does not form a gel. Therefore, it could not be involved in the analysis. Similar to the incubation at 37 °C, 90% of cells in the pellet and in SeaPrep® agarose showed no signs of compromised membrane integrity after 4 h of incubation in a noncirculating medium (Fig. 2a). However, in LEL-20, 30% of cells had compromised membrane integrity after 4 h of incubation (Fig. 2a).

Membrane integrity of HeLa cells was further tested after 4 h under bioreactor conditions (media flow 50 µl/min) using a more statistically significant and reliable method – flow cytometry. The number of cells with an intact membrane in Mebiol® gel (76%) was slightly lower, and in the case of LEL-37 (91%), it was higher in comparison with only cells on a nonadhesive surface as a control (82%) (Fig. 2b). At 20 °C in LEL-20, the number of cells with intact membranes was comparable to that in the control (~ 80%) (Fig. 2b).

**Fig. 3:**
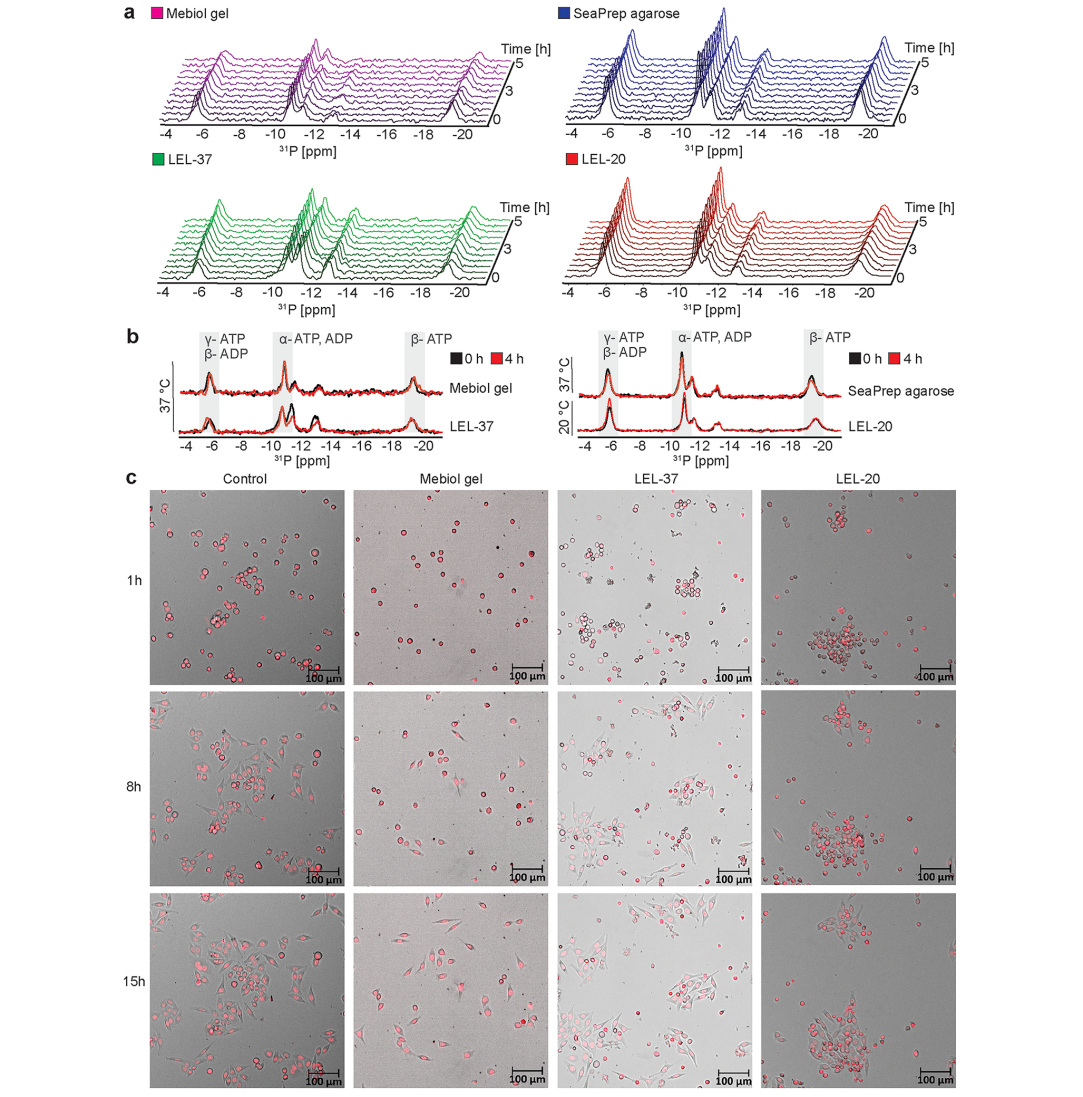
(**a**) 1D ^31^P NMR spectra indicating intracellular ATP/ADP levels of HeLa cells immobilized in SeaPrep® agarose, Mebiol® gel, copolymer LEL-37 at 37 °C and LEL-20 at 20 °C during incubation in the bioreactor at constant media flow. (**b**) 1D ^31^P NMR spectra of ATP/ADP level comparison of HeLa cells immobilized in SeaPrep® agarose, Mebiol® gel, copolymer LEL-37 at 37 °C and LEL-20 at 20 °C at the beginning of the experiment (black) and after 4 h (red) with the bioreactor system. (**c**) HeLa cells recovered from a suspension (Control), Mebiol® gel, LEL-37, and LEL-20 after 4 h of incubation with fresh media supply were plated on gelatin-coated dishes. Cell nuclei were stained with a SiR-DNA probe (red). The adhesion of cells to the surface was monitored with time-lapse microscopy

The dye exclusion-based viability assays stain the cells with the compromised cytoplasmic membrane but do not recognize the metabolic state of the cells. Intracellular levels of ATP have been previously used as a marker of cell metabolic activity during in-cell NMR experiments performed either in Mebiol® gel or agarose matrices (Kubo et al. 2013; Carvalho et al. 2019). To assess cell metabolic activity (ATP levels) in cells embedded in LEL, ^31^P NMR spectra were recorded from HeLa cells in the NMR bioreactor at a constant media flow of 50 µl/min. ATP levels were monitored as a function of time and evaluated from the intensity of the P_β_-ATP signal (~ -19 ppm). In SeaPrep® agarose, LEL-37, and LEL-20, the ^31^P NMR spectra were uniform throughout the measurement (Fig. 3a). In Mebiol® gel, a slight signal fluctuation was observed (Fig. 3a), most likely due to the mechanical instability of the sample. A comparison of cellular ATP levels at the start of measurement and after 4 h showed stable values in SeaPrep® agarose, Mebiol® gel, and LEL-37 matrices at 37 °C, as well as in LEL-20 at 20 °C (Fig. 3b). After the ^31^P NMR experiments, cells were released from the immobilization matrices and seeded on iBidi dishes coated with gelatin to investigate cell attachment to the surface. The dishes were incubated in a CO_2_ chamber at 37 °C, and confocal pictures were taken at 1 h, 8 h, and 15 h. The data showed that the cells released from Mebiol® gel and LEL copolymers were able to restore contact with the gelatin-coated surface, indicating normal cellular function and the ability to continue proliferation after the in-cell NMR measurement (Fig. 3c). Together, these data demonstrate the high survival rate of cells embedded in LEL with conditions of continuous media flow in an NMR bioreactor.

### Impact of cell immobilization in LEL on the quality of in-cell NMR spectra

**Fig. 4:**
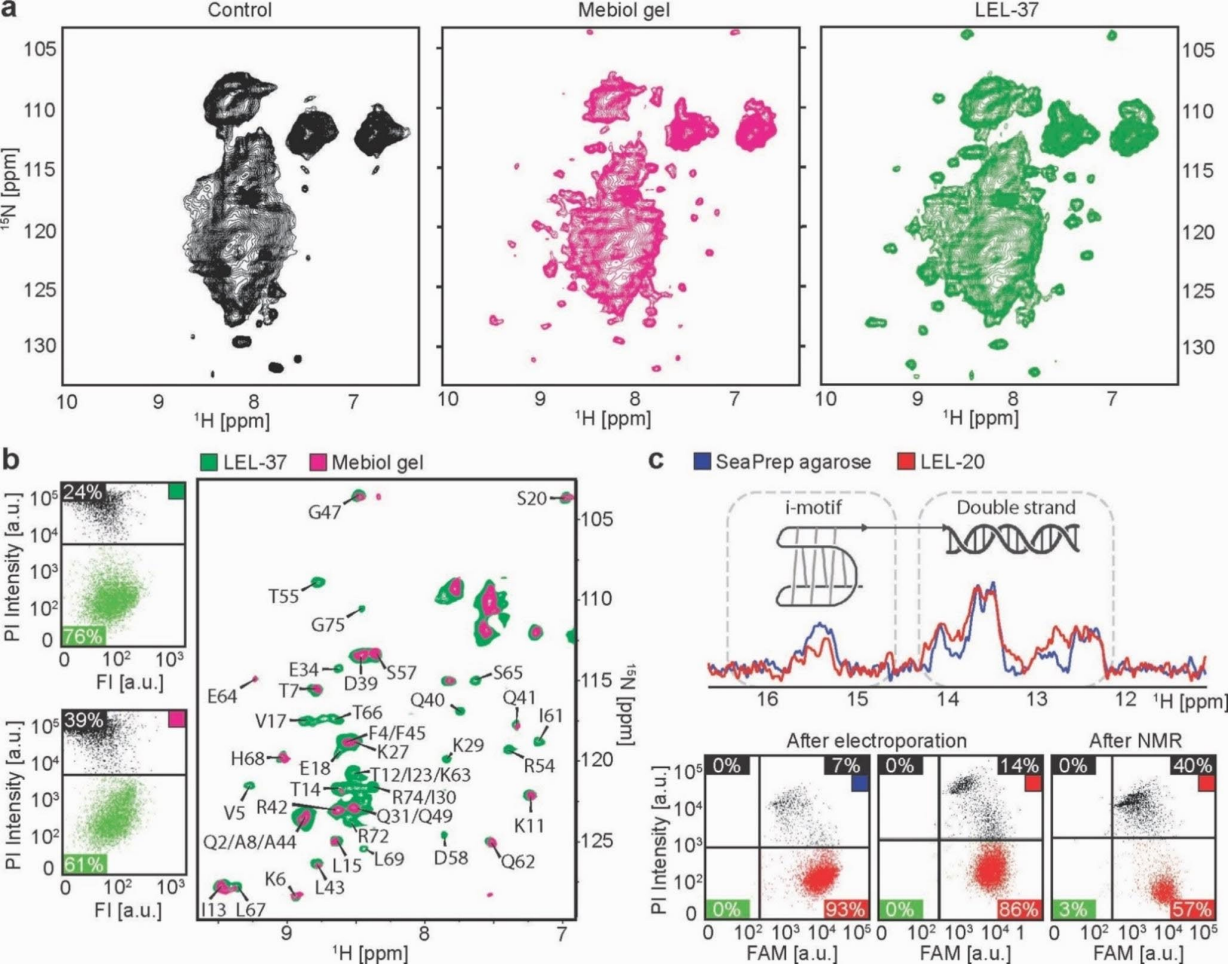
(**a**) In-cell 2D ^1^ H – ^15^ N SOFAST-HMQC spectra recorded from nontransfected HEK293T cells (Control, black), HEK293T cells transiently overexpressing ubiquitin immobilized in LEL-37 (green) and Mebiol® gel (magenta) upon 48 h incubation in ^15^ N-labeled media. (**b**) LEFT: Flow cytometry live/dead (green/black) viability assay of HEK293T cells released from LEL-37 (LEFT-UP) and Mebiol® gel (LEFT-DOWN) by propidium iodide staining after in-cell NMR measurement of cells transiently overexpressing ubiquitin protein. RIGHT: Merged in-cell NMR spectra of ubiquitin upon background subtraction; sample in LEL-37 green and Mebiol® gel magenta (assignment taken from Tanaka et al. 2019). (**c**) UP: In-cell 1D ^1^ H JR-echo spectra of HeLa cells electroporated with DNA (1:40 FAM-DNA) immobilized in LEL-20 (red) and SeaPrep® agarose (blue) measured with a bioreactor system. DOWN: Flow cytometry analysis of HeLa cell membrane integrity (PI intensity) and DNA transfection efficiency (FAM intensity). The upper parts of the diagrams show dead cells (PI positive), and the lower parts show live cells (PI negative). The left parts of the diagrams show nontransfected cells (FAM negative), and the right parts show transfected cells (FAM positive)

The performance of LEL for the bioreactor-assisted in-cell NMR experiments (relative to available gel matrices) was assessed using two model molecular systems. A protein molecule, which was represented by human ubiquitin bearing mutations L8A, I44A, and V70A that prevent the protein dimerization, previously used to determine the protein structure using in-cell NMR (Tanaka et al. 2019) and a hybrid DNA construct comprising double-stranded (ds) DNA elements and an i-motif forming segment, referred to as hybrid-ds/iM. For the protein-based target (ubiquitin), we acquired in-cell NMR spectra from samples immobilized in LEL-37 and Mebiol® gel. HEK293T cells were transiently transfected with a vector encoding human ubiquitin. After 48 h of culture in ^15^ N-labeled media, the cells were harvested and mixed with either LEL-37 or Mebiol® gel at a ratio of 1:2, and NMR samples were prepared (Supplementary Fig. S3). 2D ^1^ H-^15^ N NMR spectra of the expressed proteins were acquired for 4 h at 37 °C with a constant media flow of 50 µl/min (Fig. 4a). To overcome the problem of nonspecific peaks arising from the cellular background, nontransfected cells were incubated in the ^15^ N-labeled medium for 48 h, and the control NMR spectrum was subtracted from the experimental spectra to obtain ubiquitin-specific signals (Fig. 4b, right). Specific ^1^ H-^15^ N NMR cross-peaks for ubiquitin structure inside the cells immobilized in Mebiol® gel (reference matrix) and LEL-37 copolymer overlap at the same specific position in NMR spectra, suggesting that ubiquitin adopts the same structure in cells immobilized in LEL and Mebiol® gel. However, ubiquitin-specific signals in the in-cell NMR spectrum from cells immobilized in LEL-37 copolymer have higher intensities compared to those in the corresponding spectrum of cells immobilized in the Mebiol® gel (Supplementary Fig. S4). After in-cell NMR measurement, the cells were recovered from the samples by dissolving the gels in cold DPBS and filtering through a 70 μm strainer. Cell viability was determined with propidium iodide staining and flow cytometry analysis. The analysis revealed a higher number of cells with intact membrane integrity in LEL-37 (76%) than in Mebiol® gel (61%) (Fig. 4b, left).

LEL-20 was designed to allow in-cell NMR experiments at approximately 20 °C, which is typically used for ^1^ H NMR spectral acquisition of nucleic acids (Dzatko et al. 2018). The lower temperature of measurement for nucleic acids slows the exchange and kinetics of imino protons with water molecules from the environment (Steinert et al. 2012). Since there is no matrix currently used for in-cell NMR bioreactor experiments that form a gel at 20 °C with the ability to recover cells postmeasurement, SeaPrep® agarose was used as a reference. HeLa cells were electroporated with a DNA oligonucleotide (hybrid-ds/iM) consisting of a double-stranded region and a single-stranded C-rich overhang, which can form a pH-dependent four-stranded structure, i-motif. In 1D ^1^ H NMR spectra, peaks from 12 to 14 ppm represent signals from the double-stranded part (Watson-Crick base pairing), and peaks between 15 and 16 ppm show the presence of the i-motif structure (Fig. S5). A fraction of the electroporated DNA oligonucleotide was fluorescently labeled with FAM, which enables monitoring of the transfection efficiency. Before immobilization into SePrep® agarose and LEL-20, the samples contained 93% and 86% cells with intact membranes, respectively (Fig. 4c). 1D ^1^ H NMR spectra were recorded for 4 h. The overlay in Fig. 4c shows that the spectra from SeaPrep® agarose and LEL-20 are qualitatively comparable. After the experiment, the cells from LEL-20 were recovered, and cytoplasmic membrane integrity was analyzed with flow cytometry upon propidium iodide staining, indicating a cell survival of 57% (Fig. 4c).

## Discussion

In this study, we demonstrate that LEL is an adequate alternative to gel matrices currently used for bioreactor-assisted in-cell NMR, providing mechanical support and a favorable environment for cells in the NMR sample and having the advantage of gentle cell recovery.

LEL-20 exhibited an inverse relation of storage and loss modulus, indicating a more viscous response of the hydrogel, i.e., material behaving more as a liquid. However, the obtained values of the storage and loss modulus were still high, and no flow in the temperature range was observed using the test tube inversion method (Fig. 1b, Supplementary Fig. S3d). This phenomenon can be explained by forming multiple discrete hydrogel networks instead of one cohesive hydrogel network. These hydrogel domains are still mechanically stable but can flow to some degree. Therefore, the loss modulus has higher values than the storage modulus, but no flow is observed, as seen from the data. A further temperature increase leads to the hydrogel’s phase separation into a semipermeable micellar network and excludes water. Copolymer LEL-37 is considerably more hydrophilic due to its higher PEG content. This results in better solubility but also swelling of the copolymer and more viscous behavior at lower concentrations (Fig. 1c). Using 15 w/w %, LEL-37 undergoes phase separation above 35 °C, excluding some of the water from the hydrogel matrix, leaving phase-separated micelles. However, this copolymer cell-containing network still possesses some degree of mechanical stability (Supplementary Fig. S3c) and does not negatively influence the cell metabolic state and viability (Figs. 2 and 3). Therefore, there needs to be a compromise between the washout resistance of the immobilizer and its rheological mechanical properties.

The survival of mammalian cells embedded in agarose has been previously tested at 37 °C using trypan blue staining, parallel to protein in-cell NMR experiments (Breindel et al. 2018; Luchinat et al. 2020). Nonmanipulated HEK293T cells were almost 90% viable in agarose threads after 72 h in the bioreactor at constant media flow (Luchinat et al. 2020), and HeLa cells embedded in agarose had 99% viability after 24 h in a CO_2_ incubator (Breindel et al. 2018). With the same staining method, we have shown that more than 90% of nonmanipulated HeLa cells survive at 37 °C in SeaPrep® agarose, Mebiol® gel, and LEL-37 submersed in bioreactor medium for 4 h (duration of our demonstrative in-cell NMR spectra acquisition). Similar results were obtained at 20 °C in SeaPrep® agarose gel. However, the survival of HeLa cells in LEL-20 declined from the initial 92–69% after 4 h. This observation can be explained by the higher stiffness (1200 Pa) and concentration (25 w/w %) of LEL-20 presented in Fig. 2a, which is considerably higher than the LEL-37 counterpart (Fig. 1c), as was previously reported that high stiffness and concentration of hydrogel negatively influences cell viabilities in other hydrogel systems (Patel et al. 2014). Notably, under conditions relevant to the bioreactor-assisted NMR, i.e., under constant media flow, the survival was improved to 80%, as detected by propidium iodide staining (Fig. 2b). Moreover, cellular ATP levels remained unchanged during the same time period, indicating that cells in LEL-20 are viable and metabolically active during the bioreactor-assisted experiment (Fig. 3a and b). Obviously, cell manipulations necessary for the introduction of the studied molecules (electroporation, plasmid transfection) adversely affect cell fitness. Upon electroporation with the DNA oligonucleotide, only 60% of HeLa cells were PI negative after in-cell NMR spectra acquisition in LEL-20 (Fig. 4c), compared to 80% of PI-negative nonmanipulated cells after the same incubation in the bioreactor (Fig. 2b). Similarly, the proportion of PI-negative transiently transfected HEK293T cells overexpressing ubiquitin was 76% in LEL-37 and 61% in Mebiol® gel after a 4-hour NMR experiment (Fig. 4b), compared to 91% and 76%, respectvely, if the cells were not manipulated (Fig. 2b). This is in line with the observation of a substantial increase in PI positivity (21%) previously reported in protein-transfected HeLa cells embedded in Mebiol® gel after a bioreactor-assisted NMR experiment (Kubo et al. 2013).

Using ubiquitin as a paradigm, we demonstrated that LEL-37 is suitable for the acquisition of protein in-cell NMR spectra. In the in-cell ^1^ H-^15^ N NMR spectrum of cells overexpressing ^15^ N-labeled ubiquitin and immobilized in LEL-37, we could distinguish approximately 47 specific peaks arising from ubiquitin compared to only 25 peaks corresponding to the sample immobilized in Mebiol® gel. However, it should be noted that the positions of the peaks’ chemical shifts are overlapping and identical. The reduced signal intensities observed in the Mebiol® gel spectrum may be attributed to the lower viability of the cells (61% of live cells in the Mebiol® gel sample compared to 76% in the sample immobilized in LEL-37 (Fig. 4b) and to the mechanical instability of the Mebiol® matrix in the growth medium (Supplementary Fig. S3b). While LEL-37 threads were more rigid and stable, the threads formed from Mebiol® gel slowly dissolved and released the cells during the measurement (Supplementary Fig. S3b, S3c). This likely resulted in a lower number of cells in the NMR coil’s active area and, consequently, lower signal intensity in the case of Mebiol® gel (Supplementary Fig. S4). Since our installation of the bioreactor system doesn’t allow to control the temperature of medium on its way from a reservoir to the NMR cuvette, we can’t rule out the possibility that the gel disintegration has been caused by medium of lower temperature, entering the sample. In comparison to the agarose immobilizer, the advantage of LEL hydrogels is the ability to release the cells from the immobilizer matrix after completion of the in-cell NMR measurements, allowing their additional (biological) analysis, instead of the often tedious and time-consuming manual counting method to establish viability, as reported in previous work (Luchinat et al. 2020). 1D ^1^ H in-cell NMR spectra of DNA triplex and DNA duplex were recently acquired in a bioreactor-assisted experiment with agarose-embedded HeLa cells, thus extending the use of bioreactors for nucleic acid in-cell NMR (Sakamoto et al. 2021). Our data show that comparable in-cell NMR spectra of DNA oligonucleotides can be recorded from both samples immobilized in SeaPrep® agarose as well as in the LEL-20 matrix (Fig. 4c).

However, the copolymers used are biodegradable due to ester bond susceptibility to hydrolytic attack (Makadia and Siegel 2011). Previous reports dealing with molecularly similar PLGA-PEG-PLGA copolymers show that hydrogels were stable for at least 10–15 days before a rapid degradation took place accompanied by a significant decrease in pH (Oborná et al. 2016; Lysáková et al. 2022). As mentioned earlier, the removal of glycolic acid from the copolymer backbone leads to slower degradation due to higher hydrophobicity and thus less hydrolytic degradation (Makadia and Siegel 2011). Therefore, in terms of mechanical and chemical (pH) stability, freshly prepared LEL copolymer solutions should be suitable for bioreactor-assisted NMR measurements taking several days. However, LEL biodegradability must be considered during in-cell NMR spectra acquisition of molecules forming pH-sensitive structures (such as DNA i-motif; Fig. 4c). Nonetheless, the buffering system in DMEM was sufficient to stabilize the pH change accompanied by the evolution of lactic acid over time (Fig. 1b). In addition, the DMEM buffering system, salts, and amino acids positively influence hydrogel stiffness (López-Cano et al. 2021).

## Conclusions

In this work, we have demonstrated the suitability of PLA-PEG-PLA copolymers as immobilizers for application in bioreactor-assisted in-cell NMR. The molecular composition of LEL, i.e., the ratio of hydrophobic to hydrophilic blocks, determines its sol-gel transition temperature. Therefore, the gelation temperature can be tuned by adjusting the monomer feed ratios. The solubility of LEL-embedded samples at 4 °C allows the gentle recovery of cells for quantitative flow cytometry live/dead analyses after in-cell NMR experiments.

### Electronic supplementary material

Below is the link to the electronic supplementary material.

Supplementary Material 1
