# Supplementary material for: A thermosensitive gel matrix for bioreactor-assisted in-cell NMR of nucleic acids and proteins
Source: J Biomol NMR. 2023 Sep 9;77(5-6):203–15. doi: 10.1007/s10858-023-00422-7 (PMC10687187; doi:10.1007/s10858-023-00422-7)

**Supplementary Information**

**Title:**

**A thermosensitive gel matrix for bioreactor-assisted in-cell NMR of nucleic acids and proteins**

# Author information:

Matej Dzurov^1^, Šárka Pospíšilová^2^, Michaela Krafčíková^3,4,#^, Lukáš Trantírek^2^, Lucy Vojtová^1,*^, Jan Ryneš^2,*^

^1^ CEITEC Brno University of Technology, Purkyňova 656/123, 612 00 Brno, Czech Republic

^2^ CEITEC Masaryk University, Kamenice 753/5, 625 00 Brno, Czech Republic

^3^ National Centre for Biomolecular Research, Masaryk University, Kamenice 753/5, 625 00, Czech Republic

^4^ Institute of Biophysics, Czech Academy of Sciences, Královopolská 135, 612 65, Brno, Czech Republic

# Author identification:

Matej Dzurov <https://orcid.org/0000-0003-0620-8817>

Michaela Krafčíková [0000-0002-1210-4785](https://orcid.org/0000-0002-1210-4785)

Jan Ryneš <https://orcid.org/0000-0003-2091-390X>

Lucy Vojtová <https://orcid.org/0000-0001-5281-7045>

Lukáš Trantírek <https://orcid.org/0000-0001-5948-4837>

# ^#^ Current address: Bijvoet Centre for Biomolecular Research, Utrecht University, Padualaan 12, 3584 CH Utrecht, The Netherlands

# *Corresponding author:

[lucy.vojtova@ceitec.vutbr.cz](mailto:lucy.vojtova@ceitec.vutbr.cz), jan.rynes@ceitec.muni.cz

**Figure S1** Refractive index traces from gel permeation chromatography of LEL-20 and LEL-37 showing uniform polymer distribution.


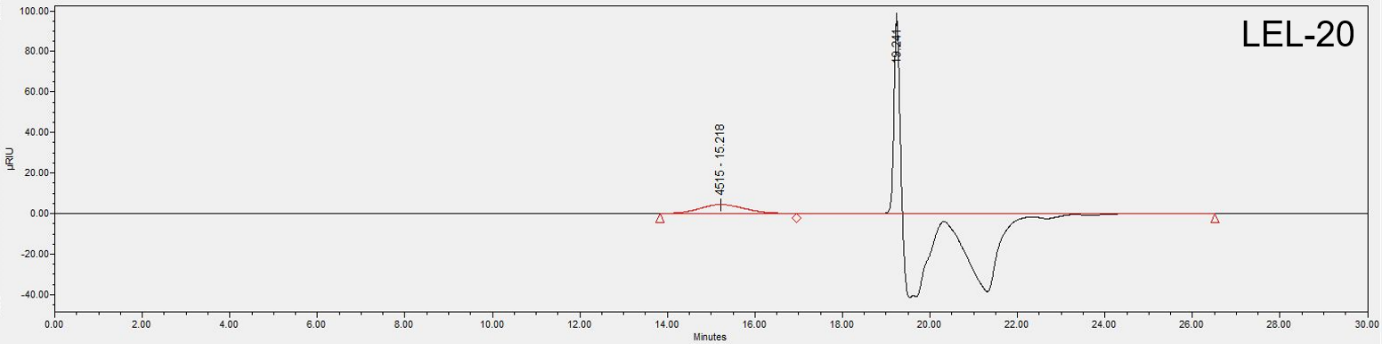


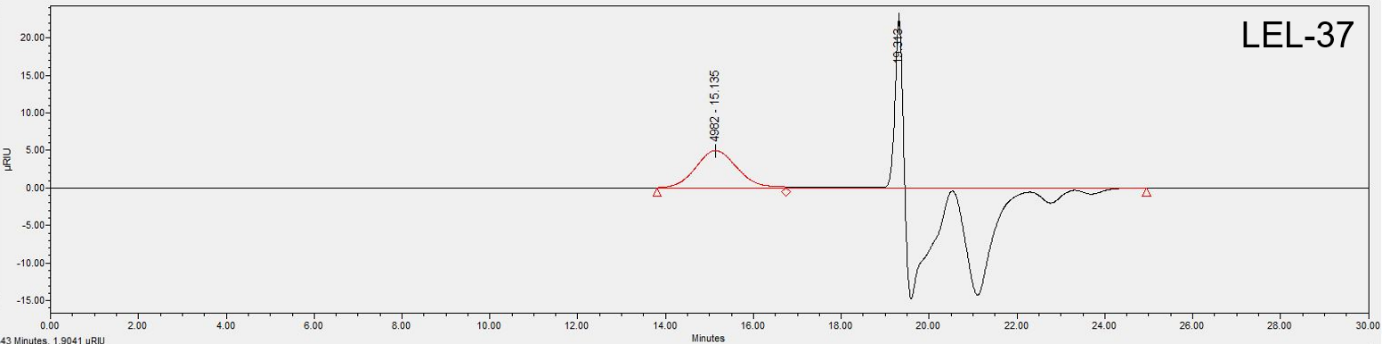


**Table S1** GPC data of LEL-20 and LEL-37 copolymers. (M_n_ – number-average molecular weight, M_w_ – weight-average molecular weight, M_P_ – molecular weight of the highest peak, PDI – polydispersity index)

| Copolymer | Retention Time [min] | M_n_ [g/mol] | M_w_ [g/mol] | M_P_ [g/mol] | PDI |
| --- | --- | --- | --- | --- | --- |
| LEL-20 | 15.218 | 3931 | 4609 | 4515 | 1.17 |
| LEL-37 | 15.135 | 4444 | 5105 | 4982 | 1.15 |

**Figure S2** 1D ^1^H NMR spectral composition of LEL-20 and LEL-37 in CDCl_3_ with assigned proton signals.


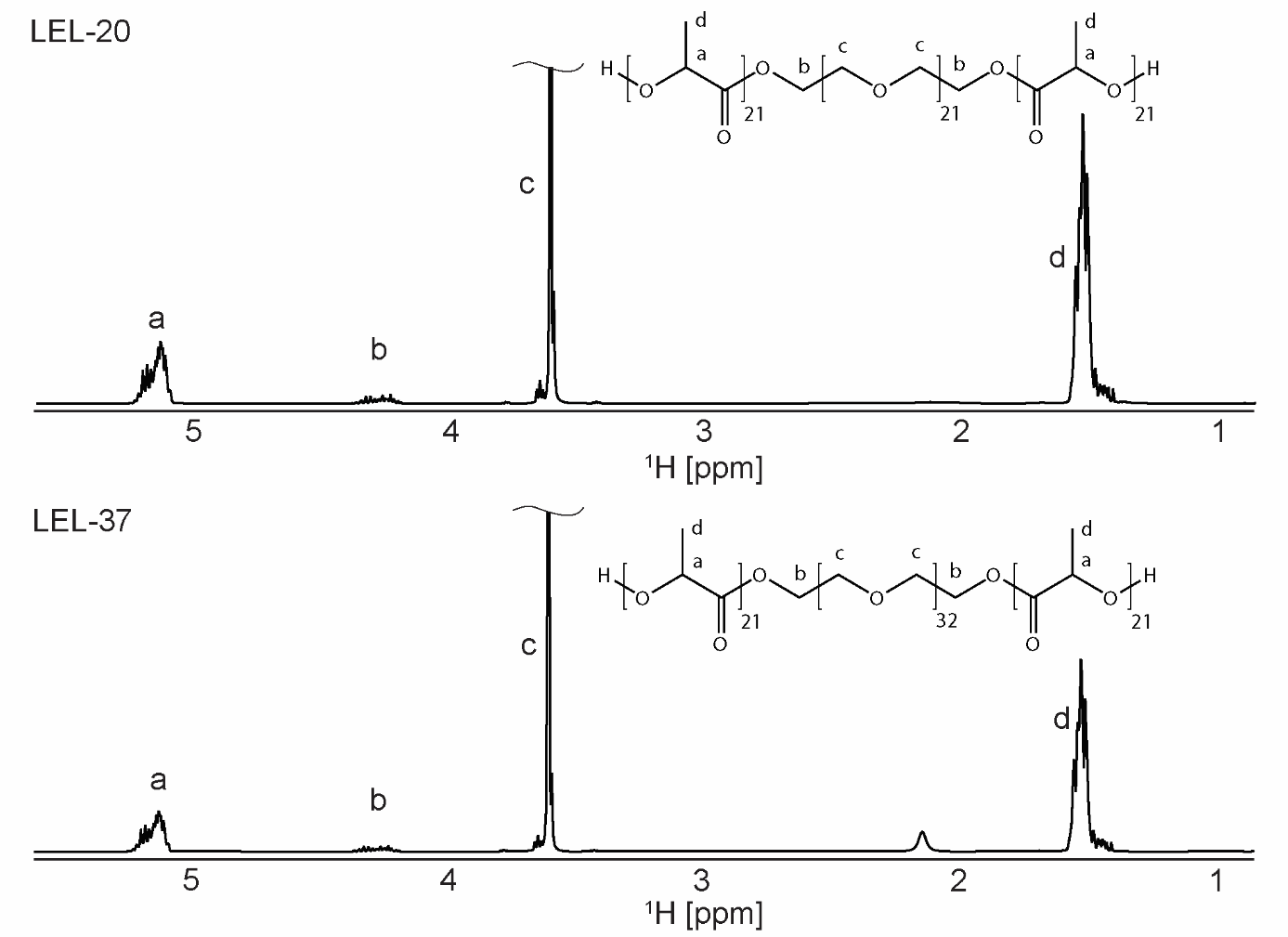


**Figure S3** Threads consisting of cells and **a)** SeaPrep^®^ agarose, **b)** Mebiol^®^ gel, **c)** copolymer LEL-37, and **d)** LEL-20, inside the 5 mm NMR tube before and after NMR measurement.


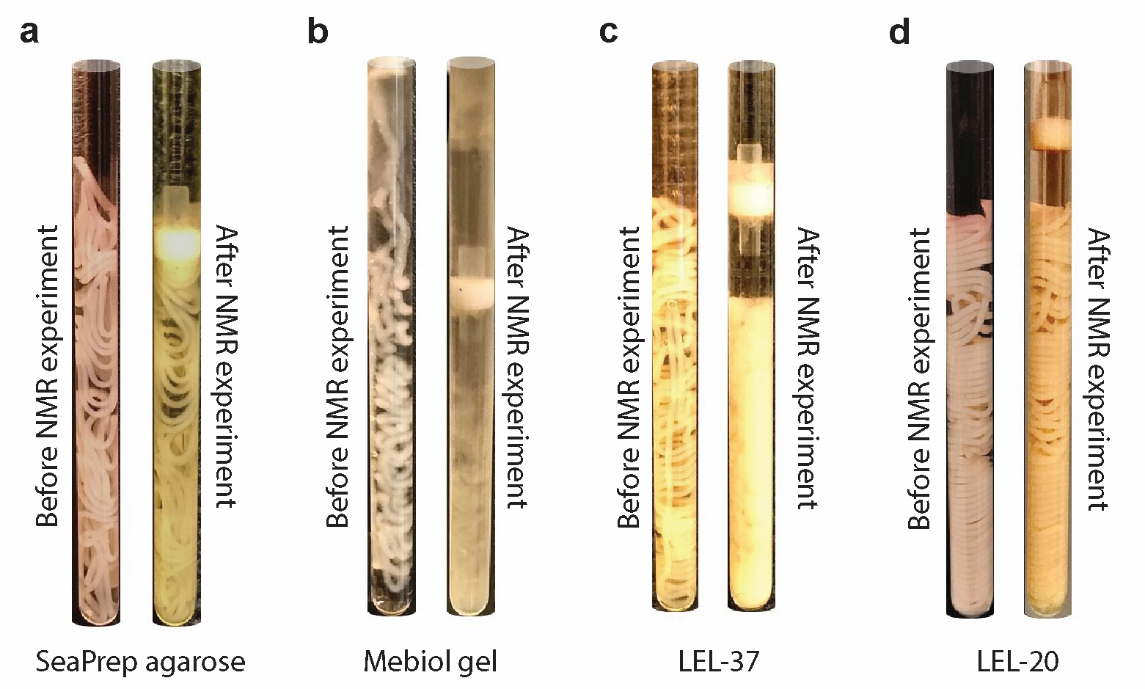


**Figure S4** 1D ^1^H projection intensity comparison from 2D ^1^H – ^15^N SOFAST-HMQC NMR spectra of cells transiently overexpressing ubiquitin protein immobilized in LEL-37 (green) and Mebiol^®^ gel (magenta) measured with a bioreactor system.


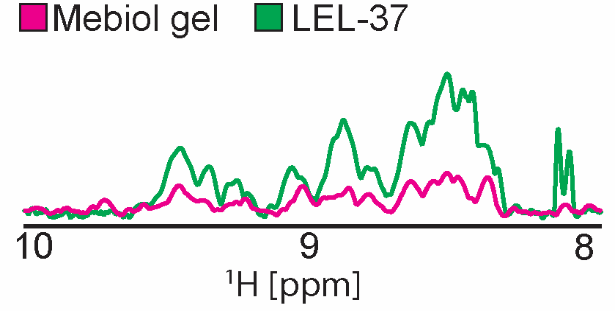


**Figure S5** In vitro 1D ^1^H NMR spectra of hybrid DNA oligonucleotide consisting of a double-stranded and i-motif region under different pH conditions.


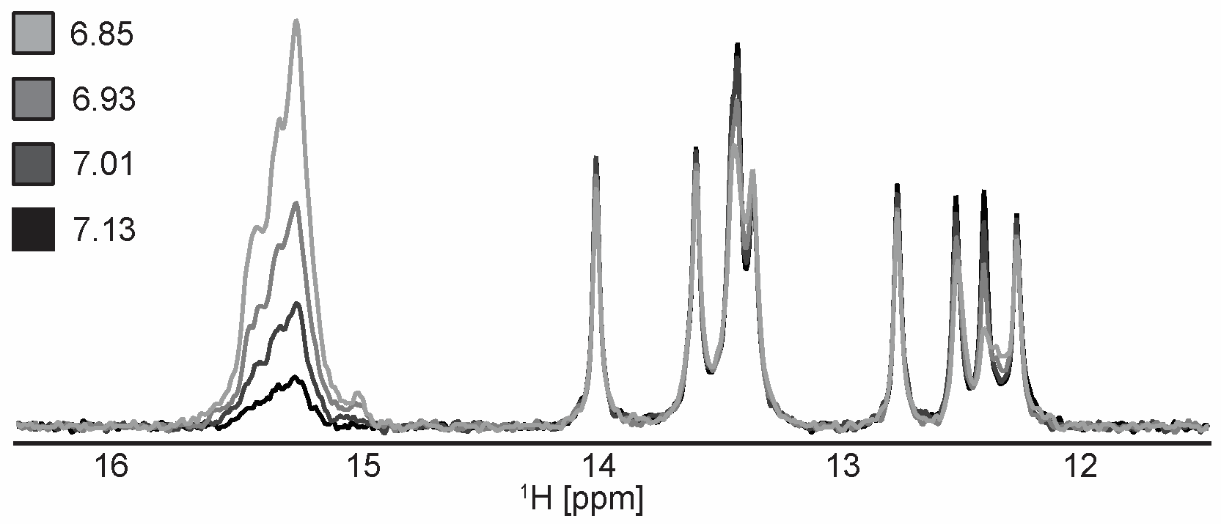

Supplement: Supplementary file 1 — Supplementary Material 1 [file 10858_2023_422_MOESM1_ESM.docx]
